# High prevalence of non-dipping patterns among Black Africans with uncontrolled hypertension: a secondary analysis of the CREOLE trial

**DOI:** 10.1186/s12872-021-02074-7

**Published:** 2021-05-22

**Authors:** Prossie Merab Ingabire, Dike B. Ojji, Brian Rayner, Elijah Ogola, Albertino Damasceno, Erika Jones, Anastase Dzudie, Okechukwu S. Ogah, Neil Poulter, Mahmoud U. Sani, Felix Ayub Barasa, Grace Shedul, John Mukisa, David Mukunya, Bonnie Wandera, Charles Batte, James Kayima, Shahiemah Pandie, Charles Kiiza Mondo, CREOLE Study Investigators

**Affiliations:** 1 St. Francis Hospital, Nsambya, Kampala, Uganda; 2 MakNCD D43 Project, Makerere University College of Health Sciences, Kampala, Uganda; 3 Faculty of Health Sciences, Busitema University, Mbale, Uganda; 4 Department of Medicine, Faculty of Clinical Sciences, University of Abuja, Abuja, Nigeria; 5 University of Abuja Teaching Hospital, Gwagwalada, Nigeria; 6 Pharmacy, University of Abuja Teaching Hospital, Gwagwalada, Abuja, Nigeria; 7 Cardiology Unit, Department of Medicine, University of Ibadan/University College Hospital, Ibadan, Nigeria; 8 Department of Medicine, Bayero University, Aminu Kano Teaching Hospital, Kano, Nigeria; 9 Division of Nephrology and Hypertension, Cape Town, South Africa; 10 Hatter Institute of Cardiovascular Research in Africa, Faculty of Health Sciences, University of Cape Town, Cape Town, South Africa; 11 Imperial Clinical Trials Unit, School of Public Health, Imperial College London, London, UK; 12 Department of Cardiology, Moi Teaching and Referral Hospital, Eldoret, Kenya; 13 Department of Clinical Medicine and Therapeutics, University of Nairobi, Nairobi, Kenya; 14 Eduardo Mondlane University Hospital, Maputo, Mozambique; 15 Douala General Hospital, Douala, Cameroon

**Keywords:** Non-dipping pattern, Dipping pattern, Uncontrolled hypertension, Black African

## Abstract

**Background:**

Dipping of blood pressure (BP) at night is a normal physiological phenomenon. However, a non-dipping pattern is associated with hypertension mediated organ damage, secondary forms of hypertension and poorer long-term outcome. Identifying a non-dipping pattern may be useful in assessing risk, aiding the decision to investigate for secondary causes, initiating treatment, assisting decisions on choice and timing of antihypertensive therapy, and intensifying salt restriction.

**Objectives:**

To estimate the prevalence and factors associated with non-dipping pattern and determine the effect of 6 months of three antihypertensive regimens on the dipping pattern among Black African hypertensive patients.

**Methods:**

This was a secondary analysis of the CREOLE Study which was a randomized, single blind, three-group trial conducted in 10 sites in 6 Sub-Saharan African countries. The participants were 721 Black African patients, aged between 30 and 79 years, with uncontrolled hypertension and a baseline 24-h ambulatory blood pressure monitoring (ABPM). Dipping was calculated from the average day and average night systolic blood pressure measures.

**Results:**

The prevalence of non-dipping pattern was 78% (564 of 721). Factors that were independently associated with non-dipping were: serum sodium > 140 mmol/l (OR = 1.72, 95% CI 1.17–2.51, *p*-value 0.005), a higher office systolic BP (OR = 1.03, 95% CI 1.01–1.05, *p*-value 0.003) and a lower office diastolic BP (OR = 0.97, 95% CI 0.95–0.99, *p*-value 0.03). Treatment allocation did not change dipping status at 6 months (McNemar’s Chi^2^ 0.71, *p*-value 0.40).

**Conclusion:**

There was a high prevalence of non-dipping among Black Africans with uncontrolled hypertension. ABPM should be considered more routinely in Black Africans with uncontrolled hypertension, if resources permit, to help personalise therapy. Further research is needed to understand the mechanisms and causes of non-dipping pattern and if targeting night-time BP improves clinical outcomes.

*Trial registration* ClinicalTrials.gov (NCT02742467).

## Background

Hypertension is the leading risk factor for mortality accounting for approximately 10 million deaths and 218 DALYs annually [1]. The prevalence of hypertension has increased in Africa from approximately 20% in 1990 to over 30% in 2010, and more than 30% of these hypertensive individuals are not aware of their diagnosis, demonstrating a huge burden of undiagnosed and uncontrolled hypertension [2, 3]. In a meta-analysis by Ataklte et al., about 18% of individuals with hypertension in Sub-Saharan Africa were receiving treatment across the studies, and only 7% had controlled blood pressure, highlighting the need for implementation of timely and appropriate strategies for diagnosis, control, and prevention [3].

Even though office blood pressure (BP) measurement has remained the standard of BP measurement globally, it gives limited information on the biological rhythms inherent to the disease process [4–6]. Ambulatory Blood Pressure monitoring (ABPM) is now the recommended method for diagnosing hypertension and assessing long term outcomes[7]. ABPM is more precise because it takes more measurements in periods containing the main sources of BP variability [6]. The mean 24-h, daytime, and night-time indicators have classically been used for both the relationship between ambulatory BP and cardiovascular prognosis, as well as for assessing the antihypertensive effect of drugs [4–6, 8–13].

Furthermore, ambulatory BP readings also provide information on the dipping status [7]. Dipping of BP in the night is a normal physiological process that is due to the reduction in sympathetic tone and the parallel increase in vagal activity during the sleep period. A reduction of > 10% to 20% in systolic and diastolic BP in the night, compared to daytime readings, is defined as normal dipping. Patients with a nocturnal reduction > 20% are defined as extreme dippers, while those with < 10% reduction BP levels are defined as non-dippers. On the other hand, reverse dippers or risers are those with a paradoxical rise in the night BP [14–16].

Non-dipping pattern is associated with hypertension mediated organ damage (HMOD), secondary forms of hypertension and poorer long-term outcomes [17–20]. Identifying a non-dipping pattern may be useful in assessing risk, aiding the decision to investigate for secondary causes, initiate treatment, assist decisions on choice and timing of antihypertensive therapy, and intensifying salt restriction.

Treatment based on dipping status is an emerging novel concept for identifying high-risk hypertensive patients who would derive maximum benefit with antihypertensive medication [21]. Unfortunately, the diagnostic and prognostic importance of dipping patterns has not been widely adopted in Sub-Saharan Africa, probably because of limited resources [22].

## Methods

### Study aims

Therefore, this study aimed to describe the dipping patterns of Black African patients with hypertension and determine whether antihypertensive therapy leads to a change of dipping status at 6 months of therapy in a post-hoc analysis of the CREOLE trial [23, 24].

### Study design

The CREOLE Trial was a multicenter, single-blind randomized trial comparing dual therapies of amlodipine plus hydrochlorothiazide, amlodipine plus perindopril and perindopril plus hydrochlorothiazide for lowering BP in Black Africans. The detailed study methods have been previously described [23, 24]. With relevant ethics approval and written informed consent from participating sites and participants, respectively, the trial was prospectively registered and updated at ClinicalTrials.gov (NCT02742467). We performed a secondary analysis of the CREOLE dataset to estimate the prevalence and factors associated with non-dipping pattern and determine the effect of 6 months of antihypertensive therapy on the dipping pattern among Black African hypertensive patients.

### Study setting

This study was conducted in 10 centers in 6 Sub-Saharan African countries between June 2017 and June 2018. The study sites were in Abuja, Ibadan and Kano in Nigeria, Cape Town, South Africa, Eldoret and Nairobi in Kenya, Maputo in Mozambique, Douala in Cameroon and Kampala in Uganda.

### The CREOLE trial (the primary study)

This was a multicenter study, whose rational and design have been previously published [23, 24]. Briefly, eligible patients were randomized to three arms of dual antihypertensive therapy: a daily regimen of 5 mg of amlodipine plus 12.5 mg of hydrochlorothiazide, 5 mg of amlodipine plus 4 mg of perindopril, or 4 mg of perindopril plus 12.5 mg of hydrochlorothiazide for 2 months. Doses were then doubled (10 and 25 mg, 10 and 8 mg, and 8 and 25 mg, respectively) for an additional 4 months.

### Study participants

Data of the 721 Black African patients with uncontrolled hypertension enrolled in the randomized trial that had a 24-h ambulatory BP measurement at baseline were analyzed in this study.

### Study procedure

Enrolled patients had a 24-h ABPM using a validated device (Meditech monitor [ABPM-05 model] and Meditech BP cuff) at the baseline and 6 months’ study visits. Patients wore the ABPM device for a minimum of 24 h with automatic readings every 30 min in the daytime and hourly at night. Daytime was defined as 9 am through 9 pm, and night-time as 12 midnight through 6 am. Small, medium or large blood pressure cuffs were utilized as appropriate on the non-dominant arm. Acceptable criteria of ABPM were 24 h measurement with at least 80% of available readings.

Patients were reviewed every 2 months but ABPM was only done at baseline and 6 months visits. Office BP was measured at every study visit.

Blood samples for serum creatinine, urea, random blood sugar, lipid profile and full blood count were collected at baseline and 6 months visits.

### Data analysis

Participants were grouped by baseline dipping category into extreme dippers, dippers, non-dippers and reverse dippers. The dipping category was assessed using the calculated difference between daytime mean systolic BP and nighttime mean systolic BP expressed as a percentage of the mean daytime systolic BP value at each visit. The dipping values were later dichotomized into non-dippers (dipping values < 10%) and dippers (dipping values ≥ 10%) at both baseline and 6 months.

Baseline continuous variables including age in years, body mass index (BMI), average office heart rate, average office systolic BP, average office diastolic BP, fasting blood glucose, serum creatinine and eGFR, serum urea, serum sodium, serum potassium, high density lipoprotein (HDL), low density lipoprotein (LDL), total cholesterol, triglycerides (TG), blood hemoglobin concentration and white blood cell count, were extracted from the primary data set and summarized as means and standard deviations.

Baseline categorical variables including gender, history of smoking, history of diabetes mellitus, history of alcohol use, duration of hypertension (below and above 1 year), age category (above and below 55 years) and treatment arm randomized to, were extracted from the primary data set and summarized as percentages.

Comparison of the participant baseline characteristics between the two dipping categories (non-dippers and dippers) was performed using T-test for the continuous variables and chi-squared test or appropriate non-parametric tests for categorical characteristics based on their distribution.

Multivariable logistic regression analysis was used to identify predictors of non-dipping status. Predictors of non-dipping status included in the final multivariable model were chosen a priori based on published literature.

To determine the effect of antihypertensive therapy on the dipping category we conducted the McNemar’s test for the patients who had both the baseline and 6 months ambulatory BP measurements done. Subgroup analysis using McNemar’s test was also conducted in the three randomized treatment arms to determine if any of the antihypertensive therapies had effect on the dipping category.

## Results

### Participant characteristics

The study cohort included 721 participants with 454 (63%) females. The mean age was 51.0 ± 10.8 years. Of the 721 participants, 371 (51%) had been diagnosed with hypertension for less than one year. Only 39 (5%) had diabetes mellitus. There were 14 (1.9%) current smokers and 160 (22%) were current users of alcohol.

### Prevalence of different dipping patterns using systolic ABPM

Among the 728 randomized patients in the trial, 721 patients had a complete ABPM at baseline and were included in the secondary analysis.

**Fig. 1:**
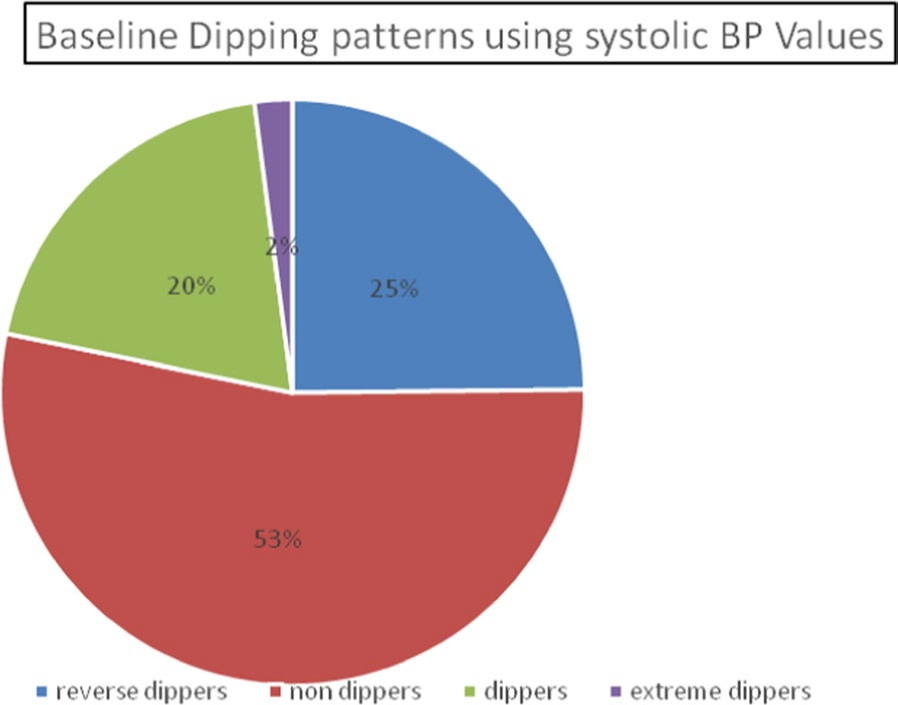
Pie chart showing the baseline systolic dipping patterns among Black African hypertensive patients in the CREOLE trial

The non-dippers accounted for 385 (53%) of the study population, reverse dippers for 179 (25%), dippers for 142 (20%), and the extreme dippers for 15 (2%). The prevalence of non-dipping (non-dippers and reverse dippers) was 78% (Fig. 1).

### Baseline characteristics among the non-dippers and the dippers

We compared the baseline characteristics of the non-dippers (non-dippers and reverse dippers, 78%) with dippers (extreme dippers and dippers, 22%) by univariate analysis (Table 1). The non-dippers were significantly older (*p*-value 0.0009); more likely to use alcohol, (*p*-value 0.008) and suffer from diabetes (*p* = 0.027). Additionally, the non-dippers had significantly higher office systolic BP (*p*-value 0.041) and serum sodium (*p*-value 0.004); and significantly lower serum haemoglobin (*p*-value 0.009).

**Table 1:** Baseline characteristics among 721 Black African hypertensive patients in the CREOLE trial based on dipping pattern

Variable	Non-dippers N = 564 (78%)	Dippers N = 157 (22%)	*P* value
Age, years	52 ± 11	48 ± 10	0.001
Gender, female	353 (63)	101 (64)	0.689
BMI^e^ kg/m^2^ < 25(normal)	152 (27)	48 (30)	
25– < 30 (overweight)	207 (37)	64 (41)	
≥ 30 (obese)	204 (36)	45 (29)	0.210
Treatment arm			
Amlodipine and hydrochlorothiazide	194 (34)	49 (31)	0.245
Amlodipine and perindopril	191 (34)	47 (30)	
Perindopril and hydrochlorothiazide	179 (32)	61 (39)	
Hypertension duration < 1 year	285 (51)	86 (55)	0.347
Diabetes mellitus	36 (6)	3 (2)	0.027
Current smoking	56 (10)	15 (10)	0.889
Current alcohol use	196 (35)	37 (24)	0.008
Office heart rate, beats/minute	82 ± 14	80 ± 13	0.148
Office systolic BP, mmHg	160 ± 11	158 ± 10	0.041
Office diastolic BP, mmHg	98 ± 10	99 ± 8	0.144
Day ambulatory systolic BP, mmHg	149 ± 1	148 ± 1	0.780
Night ambulatory systolic BP, mmHg	146 ± 1	127 ± 1	0.000
Day ambulatory diastolic BP, mmHg	91 ± 0	93 ± 1	0.091
Night ambulatory diastolic BP, mmHg	86 ± 1	76 ± 1	0.000
Serum sodium^c^, mmol/L ≤ 140	284 (51)	97 (63)	
> 140	272 (49)	58 (37)	0.011
Serum potassium^d^, mmol/L ≤ 4.0	243 (43)	66 (42)	
> 4.0	319 (57)	91 (58)	0.788
Estimated glomerular filtration rate^d^, ml/min ≥ 60	539 (96)	153 (97)	
< 60	23 (4)	4 (3)	0.480
Serum urea, mmol/L ≤ 7.1	411 (77)	128 (83)	
> 7.1	121 (23)	26 (17)	0.119
Fasting blood sugar, mmol/L	5.4 ± 1.9	5.2 ± 1.1	0.219
Low density lipoprotein^a^, mmol/L ≤ 2.6	173 (36)	53 (38)	
> 2.6	303 (64)	85 (62)	0.658
Total cholesterol, mmol/L ≥ 5.2	328 (68)	89 (64)	
> 5.2	152 (32)	51 (36)	0.291
High density lipoprotein, mmol/L ≤ 1.0	139 (29)	51 (36)	
> 1.0	341 (71)	89 (64)	0.092
Triglycerides, mmol/L ≤ 1.70	317 (66)	88 (63)	
> 1.70	162 (34)	51 (37)	0.531
Haemoglobin^b^, g/dl ≤ 12	77 (20)	14 (15)	
> 12	309 (80)	80 (85)	0.262
White blood cell count, * 10^9^/L	5.8 ± 1.8	6.1 ± 2.1	0.186

^a^107 missing values

^b^241 missing

^c^3 missing values and estimated glomerular filtration rate was calculated based on the ckd-epi creatinine equation

^d^2 missing values

^e^1 missing value

There was no difference in dipping status between males and females, in lipid profile, smoking status and kidney function.

### Predictors of non-dipping pattern among Black Africans with Hypertension

On multivariable logistic regression, only the serum sodium > 140 mmol/L, a higher office systolic BP and lower office diastolic BP were significantly associated with non-dipping pattern. Individuals with serum sodium > 140 mmol/L were 1.72 times as likely to be non-dippers as compared to dippers (95% CI 1.17–2.51, *p*-value 0.005). A unit increase in office systolic BP was associated with 1.03 times the odds of being a non-dipper as compared to being a dipper (95% CI 1.01 to 1.05, *p*-value 0.003). A unit increase in office diastolic BP was associated with 0.97 times the odds of being a non-dipper as compared to being a dipper (95% CI 0.95 to 0.99, *p*-value 0.03). There was no association with treatment allocation (Table 2).

**Table 2:** Independent predictors of non-dipping pattern after logistic regression

Variable	Adjusted odds ratio	95% CI	*p*-value
Serum sodium, mmol/L			
≤ 140	Reference		
> 140	1.72	1.17–2.51	0.005
Serum urea, mmol/L			
≤ 7.1	Reference		
> 7.1	1.56	0.95–2.54	0.076
Treatment arm			
Amlodipine plus HCTZ	Reference		
Amlodipine plus perindopril	1.13	0.71–1.81	0.598
Perindopril plus HCTZ	0.73	0.47–1.13	0.158
Age category			
≤ 55 years	Reference		
< 55 years	0.77	0.50–1.17	0.220
Office systolic BP	1.03	1.01–1.05	0.003
Office diastolic BP	0.97	0.95–0.99	0.030
Office heart rate	1.01	0.99–1.03	0.130

### Change of dipping pattern following 6 months of antihypertensive therapy

Of the 721 patients who had a complete ABPM at baseline, only 619 had a complete ABPM at 6 months. Following the antihypertensive therapy, 19% (91/487) changed from non-dippers at baseline to dippers at 6 months and 61% (80/132) changed from dippers at baseline to non-dippers at 6 months. This was not statistically significant; McNemar’s Chi^2^ 0.71, *p* value 0.400. Even after stratification in the different treatment arms there was no statistically significant change in the dipping category (Table 3).

**Table 3:** Distribution of 619 patients in the different arms according to dipping category at baseline and at 6 months

Treatment arm	Baseline dipping category		6 months dipping category	
	Non-dippers 487 (79%)	Dippers 132 (21%)	Non-dippers 476 (77%)	Dippers 143 (23%)
Amlodipine and HCTZ, n (%)	175 (36)	40 (30)	166 (35)	49 (34)
Amlodipine and PERINDOPRIL, n (%)	166 (34)	39 (30)	157 (33)	48 (34)
Perindopril and HCTZ, n (%)	146 (30)	53 (40)	153 (32)	46 (32)

McNemar’s Chi^2^ 0.71, *p* value 0.400

## Discussion

A large proportion of African hypertensive patients are non-dippers. Previous studies have reported a varying prevalence of non-dipping pattern ranging from 36 to 89% depending on the group studied [25–35]. In addition, two previous studies showed that black patients are more likely to have a higher percentage of a non-dipping pattern than patients of other races [36, 37]. Hebert et al. showed a higher prevalence of non-dipping pattern of 63% among African Americans compared to 47% in European Americans [36].

The high prevalence of a non-dipping pattern in our study may be explained by the fact that all the patients randomized had uncontrolled hypertension, as poorly treated hypertension has been linked to non-dipping pattern [34]. For example, Ben-Dov et al*.* in their study of well treated hypertensive patients found a prevalence for non-dipping pattern of 37% [30]. Second, half of our patients had been diagnosed with hypertension for more than one year compared to a study by Mitu et al*.* that enrolled newly diagnosed hypertensive patients and found a prevalence of non-dippers at 44% [38]. Non-dipping pattern is associated with a longer duration of hypertension [33], probably because the longer the duration, the higher the risk for cardiovascular disease. Third, in the study by Mitu et al*.,* patients with obstructive sleep apnea, diabetes mellitus, arrhythmias were excluded [38], yet these conditions are strongly associated with a non-dipping pattern [28, 32, 34, 35], probably accounting for the lower prevalence of non-dippers in these studies. Undiagnosed obstructive sleep apnea, diabetes mellitus and arrhythmias, may contribute to the higher prevalence of the non-dipping pattern in our patients.

Non-dipping pattern was significantly associated with serum sodium greater than 140 mmol/L. Bankir et al*.* in a large group of subjects from African descent, found that individuals who are poor day time sodium excretors have an increased night time BP and a blunted nocturnal BP dipping [39]. BP is normally lowest at night, sodium excretion is as well, but if sodium has been retained during the day, BP is adjusted to the higher level needed to eliminate it, and non-dipping results [40]. Hypertensive subjects prone to retain sodium to a degree that affects BP (salt sensitive individuals), are generally non-dippers. Black individuals with hypertension are known to be more salt sensitive than individuals of other races [41–43]. Individuals with salt sensitive hypertension have increased blood pressure levels during nighttime, where a significant sodium load is excreted following pressure-natriuresis mechanisms [44, 45]. Non-dippers retain sodium during the day, probably because of vigorous anti-natriuresis during upright activity, and excrete the excess sodium at night [40]. High intake of sodium may therefore cause non-dipping hypertension [46]. In a study by Uzu et al*.*, sodium restriction in salt sensitive hypertensive patients caused the circadian rhythm of blood pressure to shift from a non-dipping to dipping pattern [47]. Wilson et al. also showed that non-dipping pattern converted to dipping pattern after a high potassium diet in a Black population, which mitigates the effects of high sodium intake [48].

Higher serum sodium levels with non-dipping pattern suggests that there might be high prevalence of hyperaldosteronism among these uncontrolled hypertensive subjects [43]. Studies have showed an association of hyperaldosteronism with non-dipping pattern [49–51]. There is need for further research in the prevalence of hyperaldosteronism in Black African patients with uncontrolled hypertension to personalize treatment.

Office systolic BP was found to be associated with non-dipping pattern. This is similar to the study by Bochud et al*.* that assessed data from 371 individuals of African descent in the Seychelles Islands and 295 Caucasian individuals from Switzerland and found that the white-coat effect occurring in the physician’s office is associated with reduced nocturnal BP dipping in two genetically and environmentally different populations [52]. This may mean that patients with high office systolic BP may predict a non-dipping pattern. Patients with a high office systolic BP may need to do an ABPM to determine the dipping pattern.

In this study, office diastolic BP was associated with a non-dipping pattern. A higher systolic BP and lower diastolic BP may estimate a higher pulse pressure or isolated systolic hypertension. This may be comparable to a study in elderly isolated hypertensive patients that found that non-dippers had a higher average pulse pressure of 73 mmHg compared to 64 mmHg in the dippers [53]. The higher pulse pressure among non-dippers could be associated with the higher white coat effect on pulse pressure [54].

Our study findings showed that non-dipping pattern was not significantly associated with older age. This is not consistent with previous studies [30, 31, 33, 38, 55, 56]. We excluded patients with cardiovascular disease and since older age is a known cardiovascular risk factor, most of these patients could have been selected out. We also excluded patients who were on more than one hypertensive drug or who had a systolic blood pressure above 180 mmHg and this could have excluded more patients with severe cardiovascular risk and old age.

Despite some studies showing that metabolic syndrome, dyslipidemia and obesity are associated with non-dipping [30, 31, 33, 38, 57], our data did not demonstrate significant associations with body mass index, total cholesterol, low density lipoprotein and high density lipoprotein and the non-dipping pattern. This may be due to the fact that our study excluded patients with severe hypertension and cardiovascular disease who usually have dyslipidemia or metabolic syndrome.

Our data did not find any difference between the baseline dipping patterns and the dipping patterns after 6 months of antihypertensive therapy. Despite high serum sodium being associated with night nitriuresis, there was no change in non dipping percentage in the thiazide combination groups. Patients were followed up for a short period of 6 months and this may explain why there was no significant change in the dipping patterns in all three treatment arms. We were unable to determine if taking antihypertensive therapy at bedtime would have caused change of non-dippers to dippers since we did not specify what time patients took their medications. Chronotherapy of hypertension is a new therapeutic option still under study like in the TIME trial [58]. The Hygia Chronotherapy Trial, which reported that taking at least one antihypertensive medication at night instead of on awakening enhanced dipping and reduced relative cardiovascular risk by 45% [59], has concerns about study design and reporting [60, 61].

Our study results are generalizable to Black African participants with uncontrolled essential hypertension in low-income settings. We considered a single 24 h ABPM to classify the patients into non-dippers and dippers. This has been found to be moderately reproducible over time as mentioned in previous studies which recommend a 48 h ABPM [62, 63]. However, the second ABPM seems to show similar results to that at baseline. It is also important to note the patients were followed up for a short period of 6 months which made it difficult to assess for cardiovascular events and the effect of treatment on dipping status.

Other limitations; we had a rigid definition of day and night periods in our ABPM, and did not document times of day time napping (siesta), or waking up at night to pass urine (nocturia) during our study. These behavioral phenomena (siesta and nocturia) are more frequent in the studied population because of the hot climate in Sub Saharan Africa and this may account for the finding of very high non-dipping pattern and its "resistance" to pharmacologic therapy.

Afternoon nap, by virtue of its short duration, is devoid of interruptions, and thus can be used as a model for tiled, non-interrupted sleep. BP decline during the afternoon nap lowers the day time average BP and causes misclassification of patients as non-dippers [64–67].

Nocturia results in inclusion of awake BP measurements in the night-time BP measurements and thus raises the average night time BP, causing misclassification of patients as non-dippers [64, 68, 69].

Daytime and night-time physical activity levels are independently and significantly predictive of the magnitude of the nocturnal dip in BP [69, 70]. Variation in activity may confound interpretation of 24-h ABPM, and contribute to the poor reproducibility of dipper status [70].

## Conclusion

More than three quarters of the Black African patients with uncontrolled hypertension had a non-dipping pattern suggesting a high risk for cardiovascular morbidity and mortality in this cohort. High serum sodium, office systolic BP and diastolic BP were significantly associated with non-dipping pattern. There was no change in dipping pattern following 6 months of antihypertensive therapy. The study shows there is a need for increased utilization of the ABPM in Africa, especially in patients with a high serum sodium and high office systolic BP in order to identify non-dippers. Utilisation of ABPM needs to specify times of physical activity, sleep and frequency of nocturia by the participants. More studies are needed to investigate the need for personalized treatment, like salt restriction and chronotherapy, in non-dippers to convert them to dippers.

## Data Availability

The data that support the findings of this study are available on request from the corresponding author (PMI). The data are not publicly available due to information that could compromise research participant privacy.

## Abbreviations

ABPM: Ambulatory blood pressure monitoring
BMI: Body mass index
BP: Blood pressure
CREOLE: Comparison of Three Combination Therapies in Lowering Blood Pressure in Black Africans
eGFR: Estimated glomerular filtration rate
HCTZ: Hydrochlorothiazide
HDL: High density lipoprotein
LDL: Low density lipoprotein
TG: Triglycerides
